# Simulations and directed acyclic graphs explained why assortative mating biases the prenatal negative control design

**DOI:** 10.1016/j.jclinepi.2019.10.008

**Published:** 2020-02

**Authors:** Paul Madley-Dowd, Dheeraj Rai, Stanley Zammit, Jon Heron

**Affiliations:** a Centre for Academic Mental Health, Population Health Sciences, Bristol Medical School, University of Bristol, Bristol, UK; b NIHR Biomedical Research Centre, University of Bristol, Bristol, UK; c Avon and Wiltshire Partnership NHS Mental Health Trust, Bristol, UK; d MRC Centre for Neuropsychiatric Genetics and Genomics, Cardiff University, Cardiff, UK

**Keywords:** Assortative mating, Bias, Causal inference, Directed acyclic graphs, Negative control, Prenatal, Simulation

## Abstract

**Objective:**

The negative control design can be used to provide evidence for whether a prenatal exposure–outcome association occurs by in utero mechanisms. Assortative mating has been suggested to influence results from negative control designs, although how and why has not yet been adequately explained. We aimed to explain why mutual adjustment of maternal and paternal exposure in regression models can account for assortative mating.

**Study Design and Setting:**

We used directed acyclic graphs to show how bias can occur when modeling maternal and paternal effects separately. We empirically tested our claims using a simulation study. We investigated how increasing assortative mating influences the bias of effect estimates obtained from models that do and do not use a mutual adjustment strategy.

**Results:**

In models without mutual adjustment, increasing assortative mating led to increased bias in effect estimates. The maternal and paternal effect estimates were biased by each other, making the difference between them smaller than the true difference. Mutually adjusted models did not suffer from such bias.

**Conclusions:**

Mutual adjustment for maternal and paternal exposure prevents bias from assortative mating influencing the conclusions of a negative control design. We further discuss issues that mutual adjustment may not be able to resolve.

**What is new?**

**Key findings**
• In the negative control design, increasing assortative mating leads to increasing bias of effect estimates in models that do not mutually adjust for maternal and paternal exposure.

**What this adds to what was known?**
• The use of a mutual adjustment strategy to prevent bias from assortative mating has been suggested previously. We aimed to provide an accessible explanation using DAGs of how and why this strategy prevents bias.
• A simulation study shows empirically how highly correlated maternal and paternal exposure values can lead to biased estimates and erroneous conclusions.

**What is the implication and what should change now?**
• We highlight the importance of drawing conclusions from the mutually adjusted model when performing a negative control design of prenatal exposure.
• Mutual adjustment for maternal and paternal exposure cannot resolve all issues in the negative control design such as nonlinear combinatorial effects of maternal and paternal exposure.

## 1 Introduction

In biological research the negative control design is implemented to test whether factors other than the treatment of interest have led to a causal interpretation of experimental results (see [1] for examples). The design compares the magnitude of an estimate of a treatment–outcome association against the estimate of another association in which either the treatment or the outcome has been replaced with a variable such that the new association is not plausibly causal via the hypothesized mechanism. The negative control design has been adapted for use in epidemiological research where treatments are replaced by exposures [[1], [2], [3], [4]]. In this article we consider only negative control exposures (or more briefly, a negative exposure) and not negative control outcomes.

Within an experimental setting we can manipulate all independent variables and use randomization, which makes the interpretation of negative control experiments relatively simple. In contrast, when using observational data confounding factors that influence the exposure/negative exposure and the outcome may bias the association of interest (AOI) and the negative control association (NCA). It has therefore been emphasized that the AOI and the NCA should share similar confounding structures; in other words, the distribution of confounders across levels of the exposure and outcome should be similar to the distribution across levels of the negative exposure and outcome. Any biases, due to residual confounding should therefore influence the effect estimates of both associations equally. If the effect estimate of the AOI is substantially more extreme than that of the NCA then this provides evidence in favor of the association being causal. It is left to the researcher to subjectively interpret whether the size of the difference in effect sizes is clinically meaningful; bootstrapping can be used to create a confidence interval (CI) to allow for statistical testing of this difference.

Negative control designs are often used to assess whether prenatal exposures are causally related to outcomes via an in utero pathway. Here the association of maternal exposure with an outcome (the AOI) is compared with the association of the paternal exposure with the same outcome (the NCA). Early applications of the design assessed the association of maternal smoking in pregnancy on offspring low birthweight (see the commentary by Keyes et al. [5] for a brief history) while more recent examples using the Avon Longitudinal Study of Parents and Children (ALSPAC) are provided by Taylor et al. [6] and Richmond et al. [7]. These studies respectively assessed whether maternal smoking in pregnancy is associated with offspring depression and whether maternal body mass index (BMI) is associated with methylation of the offspring HIF3A gene. The paternal exposure may have an in utero effect, such as would be the case in the first example where passive smoking or smoking-related changes in sperm quality may influence offspring depression. It has been argued by Davey Smith, however, that associations arising by such different mechanisms are unlikely to be equal in magnitude to that of the association with the maternal exposure [3].

The estimates obtained from different statistical modeling approaches (described later) have previously been suggested to be influenced differently by assortative mating between parents [4]. Positive assortative mating describes the tendency for individuals to mate with a partner who has the same value of a given characteristic as themselves [8]. Negative assortative mating describes preference for mates with a differing value on the characteristic to one's own value.

Smoking [[9], [10], [11], [12]], alcohol use [9,10,[13], [14], [15], [16], [17], [18]], caffeine use [10], and BMI [[19], [20], [21]] have all been suggested to be characteristics correlated within pairs as a result of positive assortative mating; these characteristics are also commonly examined as in utero exposures in negative control designs. Exposure characteristics may be similar within a pair due to (1) mate selection based on the characteristic itself (e.g., nonsmoking individuals may limit their selection of partner to nonsmokers as they do not want to be exposed to smoke) or (2) selection based on determinants of the characteristic (e.g., age, education, and psychiatric and personality traits influence smoking behaviors and may also be selected on by individuals choosing a partner [20,[22], [23], [24], [25], [26], [27], [28]]). Evaluation of the evidence of the nature of exposure characteristics being similar between parents is beyond the scope of this study; however, we believe our work will show that the impact of scenarios (1) and (2) are similar in the context of negative control designs.

The most common approach in a negative control design of a prenatal exposure is to run three models (irrespective of additional models adjusting for potential confounders). Model 1 assesses the association between maternal exposure and outcome. Model 2 assesses the association between paternal exposure and outcome. Model 3 mutually adjusts both maternal and paternal exposure for each other. The maternal and paternal effect estimates are then compared against each other between Models 1 and 2 and also within Model 3.

The value of comparing estimates within Model 3 over comparing estimates between Models 1 and 2 is mentioned briefly in the appendices of Lipsitch et al.’s early description of the negative control design's use in epidemiology [1] and in Davey Smith's letter to the editor regarding this article [4]. Why this is the case has not been adequately demonstrated or discussed in the literature so far. In this study we aim to explain the importance of interpreting the difference in effect sizes obtained from the mutually adjusted model (Model 3) where exposure and negative exposure are influenced by assortative mating using directed acyclic graphs (DAGs) and a simulation study.

## 2 Directed acyclic graphs

We motivate the remainder of the study using an example that compares the influence of maternal smoking during pregnancy (the exposure) to the influence of paternal smoking during pregnancy (the negative exposure) on offspring intelligence quotient (IQ) score (the outcome). Fetal exposure to nicotine during pregnancy has been suggested to influence developmental processes in the brain including neurogenesis, migration, differentiation, and synaptogenesis [29]. These changes may influence the child's cognitive ability, measured using an IQ score. Such an association is likely to be heavily confounded as factors such as socioeconomic characteristics influence both smoking behaviors and cognitive development [[30], [31], [32], [33]]. A negative control design would therefore be useful to provide evidence of the causal nature of the association.

**Fig. 1:**
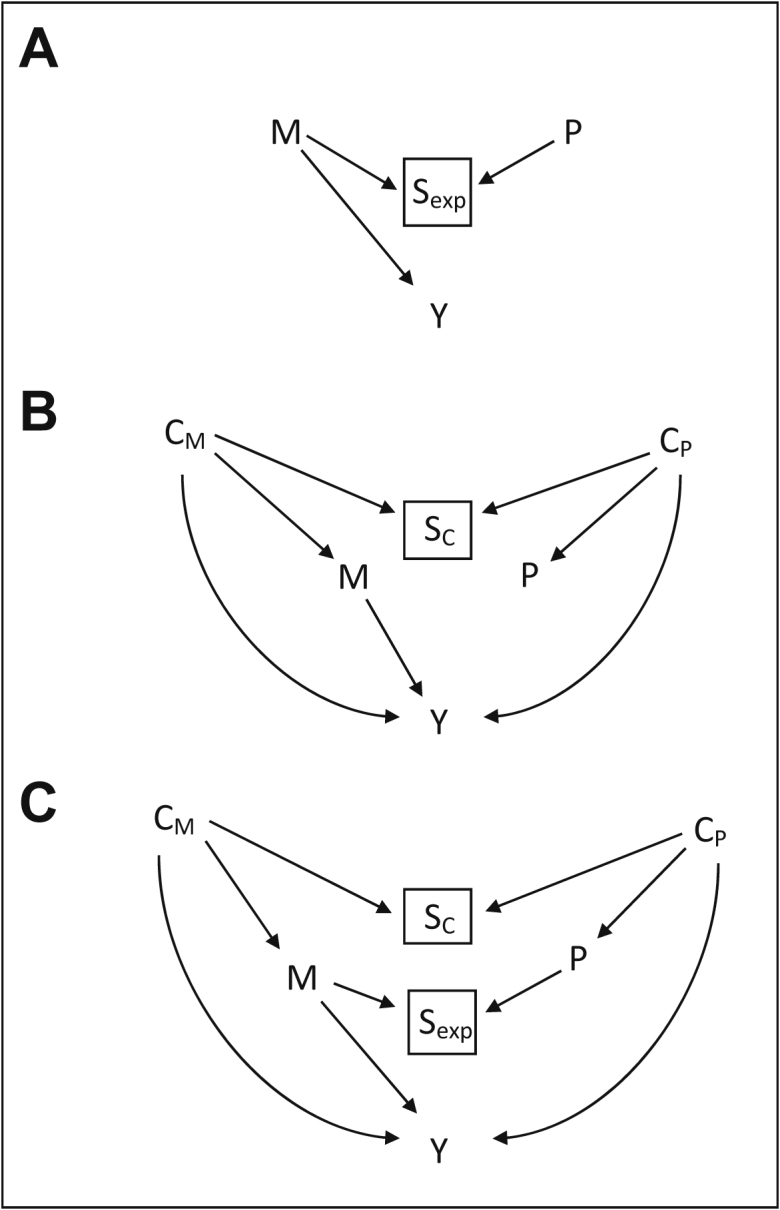
Directed acyclic graphs of the associations between variables in a negative control design with assortative behaviors. Refer to the text for descriptions of what (A), (B), and (C) represent. M is maternal smoking during pregnancy, P is paternal smoking during pregnancy, and Y is the offspring outcome. C_M_ and C_P_ are maternal- and paternal-specific confounders, respectively. S_C_ and S_exp_ are variables indicating mate selection based on confounding variables and on the exposure variable. S_C_ and S_exp_ are collider variables that when controlled for (such as when a couple have a child) induce correlation between the maternal and paternal confounders/exposures.

In Fig. 1 we show DAGs [34] of the relationship between variables in our example research question. DAGs are a useful tool to describe the causal relationship between variables and can highlight how bias is introduced, or removed, through different adjustment strategies. For those unfamiliar with DAGs, the review by Pearce and Lawlor [35] provides an accessible introduction to the concepts. In the DAGs, M is maternal smoking during pregnancy, P is paternal smoking during pregnancy, and Y is the offspring outcome. C_M_ and C_P_ are sets of confounding variables for the maternal and paternal associations with the outcome. Mate selection influenced by the exposure variable is represented by S_exp_, whereas selection influenced by confounding variables is represented by S_C_. As any of several possible mates could have been selected, we can consider each of these to be random variables. When a couple has a child together then mate selection has occurred, the couple have selected each other, and we can treat this variable as having been controlled on (represented by the box drawn around the variable). S_exp_ and S_C_ are collider variables; therefore, controlling for them will lead to correlation between maternal and paternal exposure variables and maternal and paternal confounder variables. For simplicity in our DAG we have assumed that paternal smoking during pregnancy is not causally associated with offspring outcome.

Fig. 1A shows a simplified example where the exposure behavior is selected on. Confounding is ignored in this example. Only one variable, M, directly connects to the outcome. A single backdoor pathway exists that connects P to Y (P→S_exp_←M→Y). The paternal coefficient of the paternal only model (Model 2) will be biased by the backdoor path. Mutual adjustment for M and P in a single model (as in Model 3) will close this backdoor path and eliminate the bias for the paternal coefficient. No backdoor paths exist for M. As a result, the maternal coefficient of both the maternal only model (Model 1) and the mutually adjusted model (Model 3) will be unbiased.

Fig. 1B provides an example in which the correlation between maternal and paternal exposure behavior is caused by mate selection based on determinants of these exposures, confounders for the association of maternal and paternal smoking with offspring outcome. The association of C_M_ with M and with Y is assumed to be equivalent to the association of C_P_ with P and with Y. In this example maternal and paternal smoking during pregnancy share some but not all backdoor paths to the outcome. Three variables directly connect to the outcome: M, C_M_, and C_P_. Backdoor paths along C_M_→Y and C_P_→Y exist for both M and P (e.g., M←C_M_→Y; M←C_M_→S_C_←C_P_→Y; P←C_P_→Y; P←C_P_→S_C_←C_M_→Y). An additional backdoor path exists for P that does not exist for M, via confounding variables (P←C_P_→S_C_←C_M_→M→Y). As a result, there will be additional bias for the NCA in Model 2 that will not occur for the AOI in Model 1. By mutually adjusting for M and P the additional backdoor paths for P will be closed. M and P will then have the same backdoor paths again ensuring that the biasing of the AOI and NCA are once again equivalent in Model 3.

Fig. 1C combines the examples shown in Fig. 1A and B, showing the situation in which correlation in exposure behaviors is due to selective mating based on both the exposure and the confounder variables. The backdoor paths that exist for P but not M now include both P←C_P_→S_C_←C_M_→M→Y and P→S_exp_←M→Y, which will lead to greater bias for the NCA than the AOI. Mutual adjustment for both M and P will close both of these backdoor paths leading to equivalent bias of the AOI and the NCA thereby making them comparable for the purpose of interpreting whether a causal effect may exist.

## 3 Simulation study

### 3.1 Methods

#### 3.1.1 Part 1: Simulation study of the influence of assortative mating on conclusions from the negative control design

We empirically tested how assortative mating can influence the results and conclusions from a negative control design using a simulation study. The study was motivated by the same example as for the DAG in Fig. 1A such that shared backdoor paths along confounder variables were ignored so that only the P→S_exp_←M→Y backdoor path exists.

We first simulated a binary exposure (maternal smoking in pregnancy, M), a binary negative exposure (paternal smoking in pregnancy, P), and assortative mating between the two. We simulated each exposure-pair to fall within one of the four categories of maternal and paternal smoking combinations. We fixed the prevalence of maternal smoking during pregnancy at 24% (to mimic the prevalence observed in ALSPAC) and allowed the prevalence of paternal smoking to vary across settings as we varied the extent to which smoking was assortative. Assortative mating was quantified using the pair sexual isolation index (I_PSI_, see Appendix A for formula) [36,37], a commonly used measure in evolutionary biology literature that ranges from −1 to 1. Values closer to 0 indicate no assortative mating, whereas values closer to 1 indicate a mating pair are more likely to be similar on the chosen characteristic. We investigated I_PSI_ values between 0 and 0.8, derived from the frequency in each smoking combination category (see Table 1). We did not consider negative assortative mating (I_PSI_ < 0).

**Table 1:** Frequency of observations falling into each category of maternal and paternal smoking, and the quantity of assortative mating, measured using the I_PSI_

Frequency in category (%)				I_PSI_ value (quantity of assortative mating)
1) No parent smokes	2) Mother only smokes	3) Father only smokes	3) Both parents smoke	
38.0	12.0	38.0	12.0	0.0
45.6	9.6	30.4	14.4	0.2
53.2	7.2	22.8	16.8	0.4
60.8	4.8	15.2	19.2	0.6
68.4	2.4	7.6	21.6	0.8

We then simulated a continuous outcome, which in the context of our research question we labeled “IQ score.” We simulated a normal distribution with mean 0 and variance 1 for the children who were unexposed to maternal smoking in pregnancy and a normal distribution with mean μ_M true_ and variance 1 for those exposed to maternal smoking in pregnancy. The value of μ_M true_ was varied between −5 and 5 in increments of 1. There was no effect of paternal smoking for all simulation settings.

Three regression models were fitted to the simulated data. Model 1, the maternal only model, regressed the outcome on maternal smoking only. Model 2, the paternal only model, regressed the outcome on paternal smoking only. Finally Model 3, the mutually adjusted model, regressed the outcome on both maternal and paternal smoking. We calculated the difference between β_M_ and β_P_, the coefficients for maternal and paternal smoking, between Model 1 and 2 and again within Model 3. CIs for these differences were produced using bootstrapping with 1,000 replications.

Across 1,000 simulations we investigated sample sizes of 100, 1,000, 10,000. We measured the average bias of β_M_ and β_P_ and their Monte-Carlo standard error across simulations using the *simsum* command in Stata [38]. We calculated the average difference between β_M_ and β_P_, as well as the average lower and upper bound of the CI, across simulations.

We repeated our simulation study using a binary outcome. The findings did not differ substantially from those for a continuous outcome and are presented in Appendix B.

#### 3.1.2 Part 2: simulation study of a negative control design with assortative mating where the negative exposure influences the outcome independently of the exposure

In part 1 of the simulation study we have assumed that the negative exposure has no influence on the outcome. For some exposures the negative exposure may have an independent effect on the outcome. For example, paternal smoking may influence offspring neurodevelopment through a prenatal effect (reduced sperm quality), antenatal effect (exposing the mother to smoke), or a postnatal effect (exposing the offspring to smoke). We therefore investigated how this scenario would influence the estimates of each model in the presence of assortative mating.

We repeated the simulation study, this time including an association between paternal exposure to smoking and the outcome. The outcome for this analysis was generated by simulating normal distributions (all with variance 1) with mean 0 for children who were unexposed to maternal or paternal smoking in pregnancy, mean μ_m true_ for those exposed to maternal but not paternal smoking in pregnancy, mean 2 for those exposed to paternal but not maternal smoking in pregnancy and mean μ_m true_+ 2 for those exposed to maternal and paternal smoking in pregnancy. Paternal smoking increased the outcome score by a value of 2 for all simulation settings and, as before, the value of μ_m true_ was varied between −5 and 5 in increments of 1.

### 3.2 Results

#### 3.2.1 Part 1: simulation study of the influence of assortative mating on conclusions from the negative control design

**Fig. 2:**
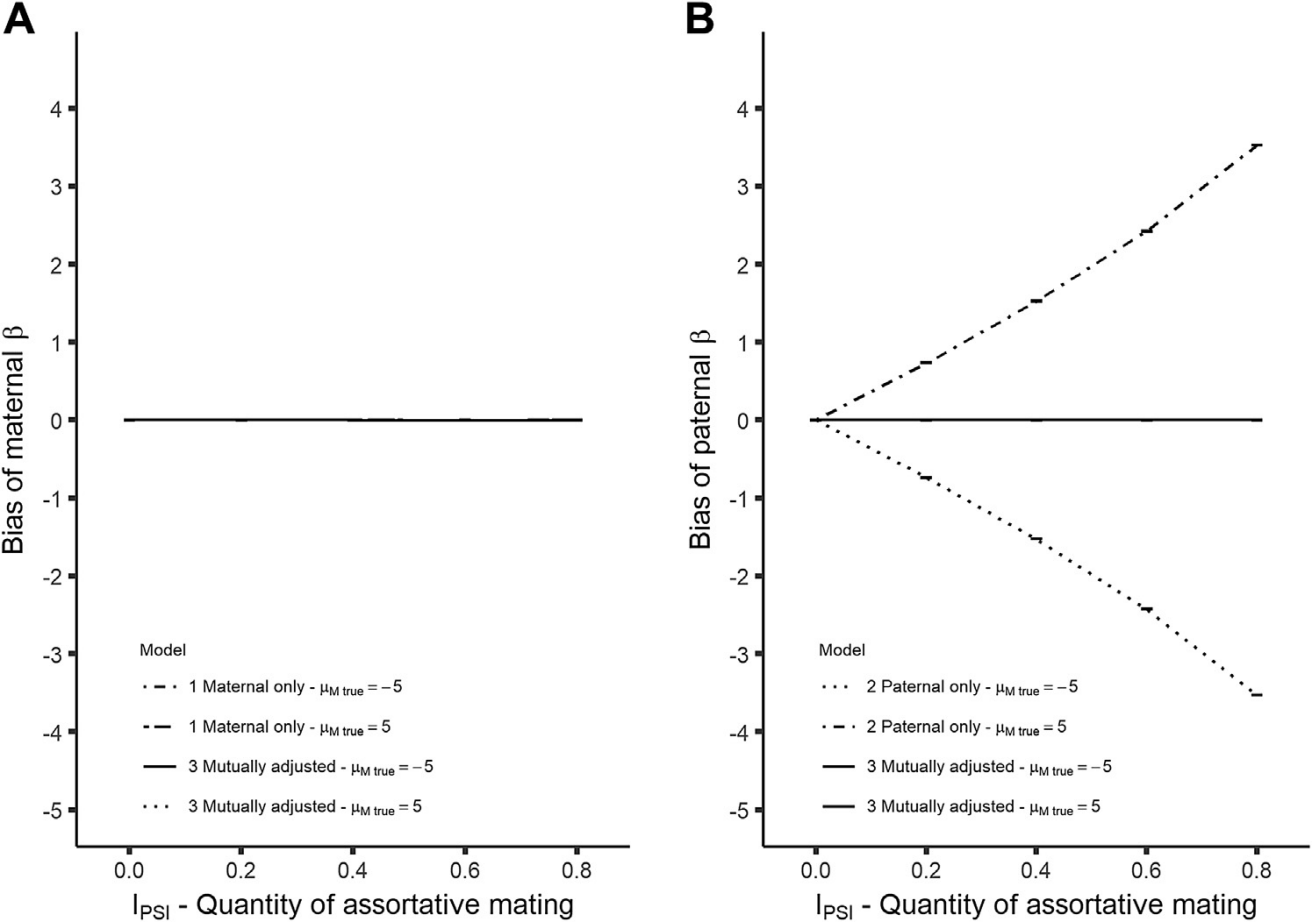
Plots of bias against quantity of assortative behavior for continuous outcome data for (A) the maternal coefficient and (B) the paternal coefficient. Error bars are 95% Monte-Carlo CIs across simulations. Sample size for data shown is 10,000. Note the large difference in Y-axis scale between the two plots.

The bias of coefficient estimates against I_PSI_ is displayed in Fig. 2. Part (A) of the figure shows that the maternal coefficient is unbiased in both Models 1 (maternal only model) and 3 (mutually adjusted model) for all quantities of assortative mating. This is true for positive and negative μ_M true_ values. Part (B) of the figure shows there is no bias for the paternal coefficient in Model 3, but there is increasing absolute bias for Model 2 (paternal only model) with increasing assortative mating. No bias is observed at an I_PSI_ of 0. This represents the case where S_exp_ does not exist and so there is no backdoor path along P→S_exp_←M→Y.

**Fig. 3:**
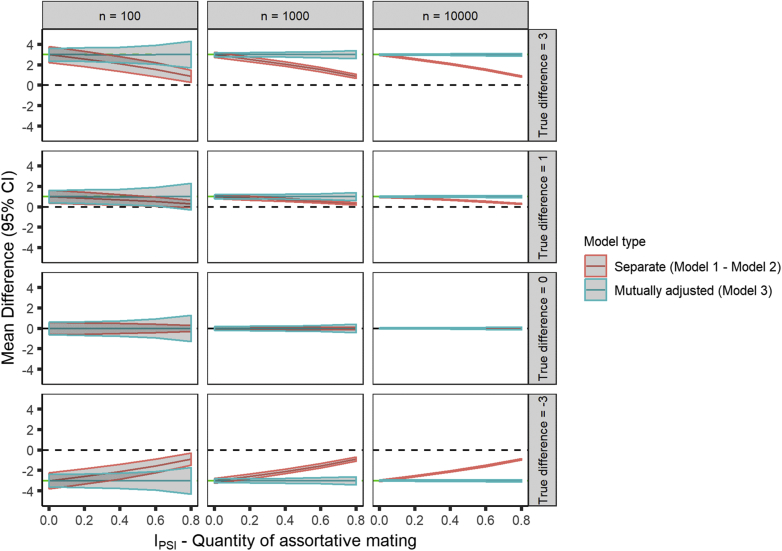
Plot of the mean difference across simulations of maternal and paternal β coefficients against the quantity of assortative mating. 95% confidence bands are the mean lower and upper CI for the difference, produced using bootstrapping. We present the difference between the coefficients of the maternal and paternal only models (red band) and the mutually adjusted model (blue band) for sample sizes of 100, 1,000, and 10,000. (For interpretation of the references to color in this figure legend, the reader is referred to the Web version of this article.)

A designed increase in the outcome in response to maternal smoking led to positive bias of the paternal coefficient in Model 2, whereas a designed decrease in the outcome in response to maternal smoking led to negative bias of the paternal coefficient. As a result, the modeled difference between the maternal and paternal coefficients from Models 1 and 2 would be smaller than the true difference when assortative mating occurs. We show this empirically in Fig. 3 where we display the mean difference across simulations (and corresponding mean 95% CI for this difference) between the maternal and paternal coefficient against the I_PSI_ for different samples and effect sizes. As the quantity of assortative mating increased the difference in coefficients between Models 1 and 2 tended toward 0. The difference in coefficients within Model 3 was unaffected by assortative mating and accurately estimated the true difference.

As the quantity of assortative mating increases the collinearity between the maternal and paternal coefficient within Model 3 increases also. This can be problematic, particularly when the sample size is small. In Fig. 3 the width of the CI for the difference between coefficients within Model 3 becomes larger with increasing assortative mating. For small effect sizes this could lead to the conclusion of a null difference when one in fact does exist (see row 2, column 1 of the figure, which shows a sample size of 100 and true difference of 1).

#### 3.2.2 Part 2: simulation study of a negative control design with assortative mating where the negative exposure influences the outcome independently of the exposure

In part 2 of the simulation study we consider a scenario in which there is an independent effect of paternal smoking in pregnancy on the outcome. Again, this example is like that displayed in Fig. 1A, but with an additional arrow from P to Y. The AOI and NCA now have the same backdoor paths, but where before only the NCA was biased by the effect size of the AOI, now both the AOI and NCA will be biased by each other where there is assortative mating in the exposure/negative exposure. Mutual adjustment for M and P can again eliminate this bias (but not bias by unadjusted confounding structures).

**Fig. 4:**
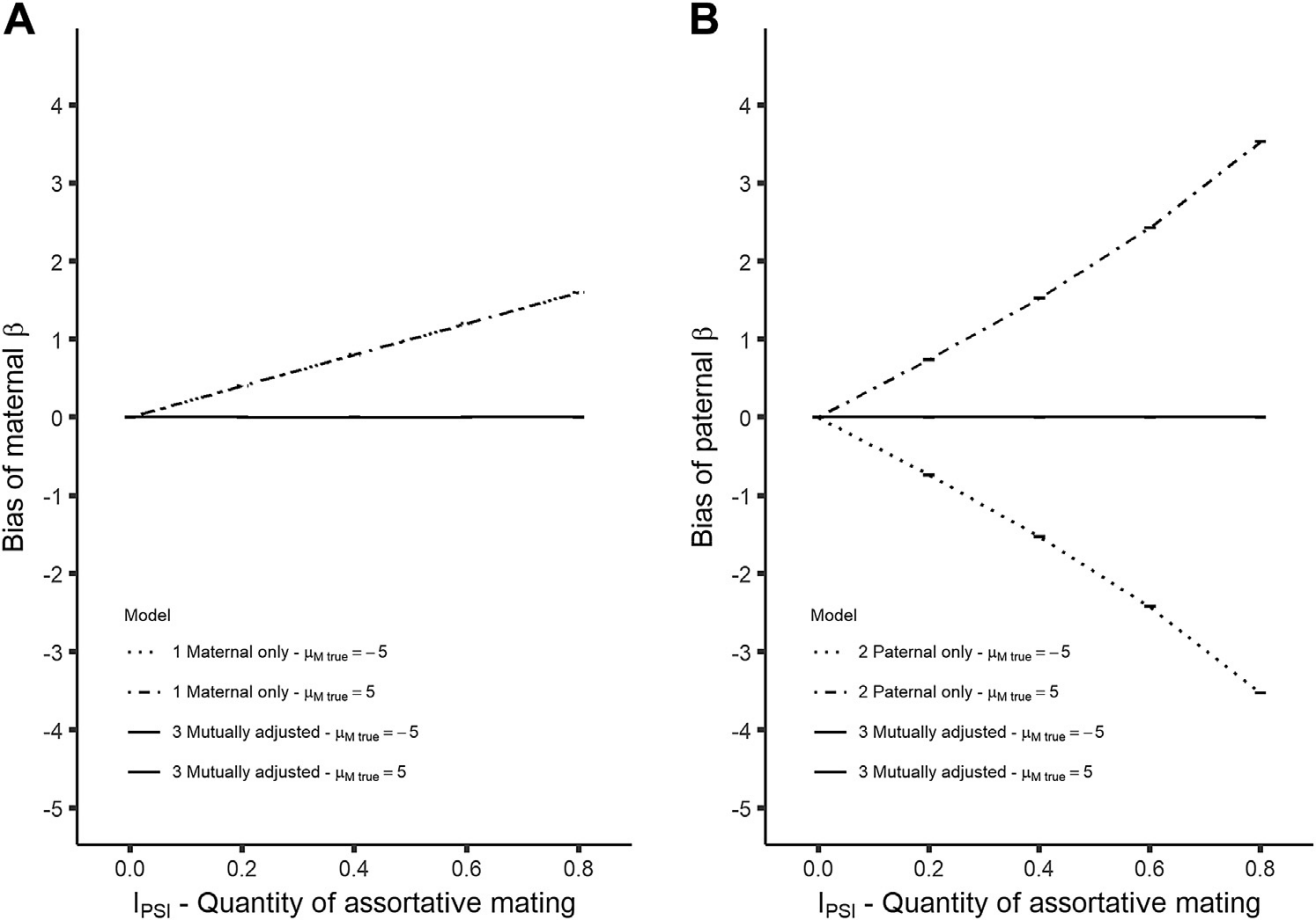
Plots of bias against quantity of assortative mating for continuous outcome data with a maternal and paternal effect for (A) the maternal coefficient and (B) the paternal coefficient. Error bars are 95% Monte-Carlo CIs across simulations. Sample size for data shown is 10,000.

In our simulations, we show that the introduction of a paternal effect leads to bias in the maternal coefficient in the presence of assortative mating for models that do not use mutual adjustment (see Fig. 4). The bias increases with increasing assortative mating. Bias in maternal β is the same for true maternal effect size of −5 as it is for +5 while the bias in the paternal β appears unchanged compared with that of the data where there is no paternal effect. This suggests that the size and direction of bias for each coefficient is dependent on the size and direction of the effect size of the other coefficient and not on the coefficient's own effect size. Models with mutual adjustment display no bias for either estimate in any setting.

Despite the introduction of bias to the maternal coefficient for the maternal only model (data in part 2) compared with data where there is no paternal effect on the outcome (data in part 1), there was little change in the pattern of results for the difference in coefficients between the data in parts 1 and 2. Supplementary Fig. C1 (see Appendix C) shows the mean difference across simulations between the maternal and paternal coefficient against the I_PSI_ for the data in part 2. Comparison with Fig. 3 shows very similar findings. This suggests that conclusions drawn from the maternal only and paternal only models will be influenced similarly by assortative mating in data where there is a paternal effect (NCA present) and where there is no paternal effect (null NCA).

## 4 Discussion

In the negative control design, correlation between the exposure and negative exposure as a result of assortative mating leads to biased effect estimates where the two exposures have not been mutually adjusted for one another. The effect estimate of one exposure is biased by the “other” exposure (i.e., the effect size of the AOI leads to bias in the effect estimate of the NCA and vice versa). Assortative mating can therefore lead to more similar effect estimates between the exposure and negative exposure. This may lead to the erroneous conclusion that there is no causal effect when one may exist. Mutual adjustment resolves this by blocking the backdoor pathway that exists via the “other” exposure. However, when the quantity of assortative mating is high the strong correlation between exposure and negative exposure leads to large standard errors for mutually adjusted model coefficients, particularly when the sample size is small. This makes the size of the difference between the AOI and NCA more ambiguous by enlarging the CI.

An important assumption of the negative control design is that the confounding structure of the AOI is equivalent or shared with that of the NCA. It is also important to remember that confounders that have not been accounted for in models or that have not been well measured will still lead to bias. An alternative approach to dealing with assortative mating has been suggested in which the father's association is modeled only in families where the mother does not smoke [39]. A possible pitfall of this approach is that it may change the distribution of confounding factors across levels of maternal and paternal smoking behavior in the data set used for analysis, leading to bias even after mutual adjustment. As the AOI and NCA would no longer share the same confounding structure the two associations would be biased to different extents by confounders and so comparison of the two may not be useful. We would argue that mutual adjustment in a dataset that includes all families is a better strategy as it maintains equivalent confounding structures while blocking backdoor paths resulting from assortative mating.

Mutual adjustment is not able to resolve nonlinear combinatory effects of exposure and negative exposure. There is evidence for differences in the smoking behaviors between couples who are concordant and discordant for smoking during pregnancy [12]. Concordant couples are likely to smoke when their partner is present while the smoking partner in a discordant couple is likely to smoke more cigarettes per day than in concordant couples. This is not something we have assessed in our study as we have only used a binary measure of smoking which would not have the ability to capture quantity of smoking. The influence on risk of outcome when using such a binary variable may therefore not be accurately represented by a model using a simple linear combination of maternal and paternal effect, as is done in the mutually adjusted model. It may be better to use categories of smoking concordance between parents (equivalent to using an interaction term between exposure and negative exposure) to account for nonlinear combinatory effects. However, if these categories are different to one another in underlying confounding structure then the negative control design may not be appropriate for this research question.

For simplicity we did not include confounding variables in the simulations. In Appendix D we present an applied example depicting how we would undertake an investigation into the association between maternal smoking during pregnancy and offspring intellectual disability in ALSPAC. Here we describe how correlation between maternal and paternal confounders may allow for using maternal confounding variables as proxies for paternal values. To our knowledge the influence of this adjustment strategy on the bias of maternal and paternal association estimates has not been tested. It is possible that adjustment for maternal but not paternal confounding variables may result in the NCA containing more bias than the AOI. Inclusion of confounding variables into the simulation study would have provided the opportunity to investigate whether imbalanced adjustment of maternal and paternal confounding variables influences the bias of point estimates.

We also did not consider the influence of measurement error in our DAGs or simulation study. This is a pertinent issue as in some cohorts the mother provides information on both her own and her partners exposure; the latter may suffer more from measurement error. Sanderson et al. [40] have shown that measurement error in the exposure or negative exposure will lead to biased effect estimates. In Appendix E we explored how the introduction of measurement error to the negative exposure variable can influence the conclusions of a negative control study in the context of an exposure affected by assortative mating. Briefly, error in a binary negative exposure can lead to bias by artificially increasing or decreasing the correlation between the exposure and negative exposure.

## 5 Conclusion

When performing a negative control study in the presence of assortative mating, the estimates used for interpretation should be those of the mutually adjusted model, although this will not resolve all issues of the negative control design. We suggest that a literature review be performed before carrying out a negative control study to assess whether the exposure, negative exposure, and relevant determinant variables may be involved in mate selection.

It is important to remember that the negative control design cannot be used to infer causality on its own. Single studies are prone to unusual and nonreplicable results. Hence causality, or the lack thereof, can only be asserted through triangulation of evidence using several different causal inference approaches. For studies of prenatal exposure these could include Mendelian randomization [[41], [42], [43]] and sibling design studies [[44], [45], [46]].

## CRediT authorship contribution statement

**Paul Madley-Dowd:** Conceptualization, Methodology, Software, Formal analysis, Investigation, Writing - original draft, Visualization. **Dheeraj Rai:** Conceptualization, Methodology, Writing - review & editing, Supervision. **Stanley Zammit:** Conceptualization, Methodology, Writing - review & editing, Supervision. **Jon Heron:** Conceptualization, Methodology, Writing - review & editing, Supervision.
