# Supplementary material for: Simulations and directed acyclic graphs explained why assortative mating biases the prenatal negative control design
Source: J Clin Epidemiol. 2020 Feb;118:9–17. doi: 10.1016/j.jclinepi.2019.10.008 (PMC7001034; doi:10.1016/j.jclinepi.2019.10.008)
Supplement: Supplementary file 1 — Supplementary Material [file mmc1.docx]

Supplementary material

Mutual adjustment reduces assortative mating bias in the negative control design assessing a prenatal exposure

Contents

[Appendix A: calculation of the pair sexual isolation index 2](#_Toc21094924)

[Appendix B: repetition of the simulation study using a binary outcome 4](#_Toc21094925)

[Methods 4](#_Toc21094926)

[Results 4](#_Toc21094927)

[Appendix C: supplementary figures 8](#_Toc21094928)

[Appendix D: applied example of maternal smoking during pregnancy and offspring intellectual disability in the Avon Longitudinal Study of Parents and Children 9](#_Toc21094929)

[Appendix E: simulation study of a negative control design with assortative mating and error in the negative exposure 12](#_Toc21094930)

[Methods 12](#_Toc21094931)

[Results 12](#_Toc21094932)

[Discussion 12](#_Toc21094933)

[Supplementary material references 15](#_Toc21094934)

# Appendix A: calculation of the pair sexual isolation index

The pair sexual isolation index (I_PSI_) [1, 2] is calculated as

$$I_{PSI}=\frac{\left( PSI_{aa}-PSI_{ab}-PSI_{ba}+PSI_{bb} \right)}{\left( PSI_{aa}+PSI_{ab}+PSI_{ba}+PSI_{bb} \right)}$$

Where

$PSI_{aa}=\frac{\left( aa \right)t}{\left( aa+ab \right)\left( aa+ba \right)}$,

$PSI_{ab}=\frac{\left( ab \right)t}{\left( aa+ab \right)\left( ab+bb \right)}$,

$PSI_{ba}=\frac{\left( ba \right)t}{\left( aa+ba \right)\left( ba+bb \right)}$,

$PSI_{bb}=\frac{\left( bb \right)t}{\left( ba+bb \right)\left( ab+bb \right)}$,

and the frequency values aa, ab, ba and bb are taken from the cells in Table A1. We present the frequencies and derivations used to calculate the I_PSI_ for each level of assortative mating in the simulation study in Table A2.

Table A1: Frequency values used to calculate the I_PSI_ statistic.

|  |  | Maternal value | |  |
| --- | --- | --- | --- | --- |
|  |  | Non-smoker | Smoker | Total |
| Paternal value | Non-smoker | aa | ab | aa + ab |
|  | Smoker | ba | bb | ba + bb |
|  | Total | aa + ba | ab + bb | t |

Table A2: Values of each stage of the I_PSI_ statistic calculation for each level of assortative mating.

| aa | ab | ba | bb | t | aa + ab | ba + bb | aa + ba | ab + bb | PSI_aa_ | PSI_ab_ | PSI_ba_ | PSI_bb_ | I_PSI_ numerator | I_PSI_ denominator | I_PSI_ |
| --- | --- | --- | --- | --- | --- | --- | --- | --- | --- | --- | --- | --- | --- | --- | --- |
| 38 | 12 | 38 | 12 | 100 | 50 | 50 | 76 | 24 | 1 | 1 | 1 | 1 | 0 | 4 | 0 |
| 45.6 | 9.6 | 30.4 | 14.4 | 100 | 55.2 | 44.8 | 76 | 24 | 1.086957 | 0.724638 | 0.892857 | 1.339286 | 0.808747412 | 4.04373706 | 0.2 |
| 53.2 | 7.2 | 22.8 | 16.8 | 100 | 60.4 | 39.6 | 76 | 24 | 1.15894 | 0.496689 | 0.757576 | 1.767677 | 1.672352666 | 4.180881664 | 0.4 |
| 60.8 | 4.8 | 15.2 | 19.2 | 100 | 65.6 | 34.4 | 76 | 24 | 1.219512 | 0.304878 | 0.581395 | 2.325581 | 2.658820193 | 4.431366988 | 0.6 |
| 68.4 | 2.4 | 7.6 | 21.6 | 100 | 70.8 | 29.2 | 76 | 24 | 1.271186 | 0.141243 | 0.342466 | 3.082192 | 3.86966953 | 4.837086913 | 0.8 |

# Appendix B: repetition of the simulation study using a binary outcome

## Methods

Exposure information was derived in the same way as for the continuous outcome. A binary outcome, Y, was then created with prevalence close to 10%. We designed the outcome to have an association with the maternal smoking value, but not the paternal smoking value. We tested designed maternal smoking odds ratio (DMOR) values between 0.5 and 3 in 0.25 increments.

For each observation, $i,$ we derived

$$\alpha_{i}= \frac{exp\left( \beta_{0}+\beta_{m true}M_{i} \right)}{exp\left( \beta_{0}+\beta_{m true}M_{i} \right)+1},$$

such that $\beta_{m true}$ was equal to the log of the DMOR value and $\beta_{0}$ was a constant coefficient equal to -log(0.9/0.1). A random uniform variable,$\tau$, between 0 and 1 was drawn for each observation. If $\tau_{i}<\alpha_{i}$ then the observation was defined as having the outcome (i.e. $Y_{i}=1)$ otherwise the observation did not have the outcome (i.e. $Y_{i}=0)$.

We then produced the same models as for the continuous outcome but instead used logistic regression. We used sample sizes of 1 000, and 10 000 as samples of 100 led to model convergence issues. The same performance statistics as for the continuous outcome were used with one exception. For the linear models we took the mean difference in maternal and paternal coefficients over simulations. For the logistic models we instead provide the ratio of the maternal OR to the paternal OR for exposure to smoking in pregnancy. To produce this statistic, we took the mean of the difference in maternal and paternal coefficients over simulations on the log scale, as these were normally distributed, and then exponentiated to provide the ratio of ORs. As the paternal OR is equal to 1 the true value of the ratio of ORs will always be equal to the DMOR. 95% confidence intervals were also created using bootstrapping of the difference on the log scale. The bounds of the confidence interval were averaged over simulations and then exponentiated.

## Results

The bias of coefficient estimates against the quantity of assortative mating for logistic regression models are displayed in Figure B1. As for the linear models the maternal coefficient is unbiased in both the maternal only model and the mutually adjusted model for all quantities of assortative mating (see part (i) of the figure). This is true for DMOR values that had positive and negative associations with the outcome. There is no bias for the paternal coefficient in the mutually adjusted model but there is increasing absolute bias for the paternal only model with increasing assortative mating (see part (ii) of the figure).

Figure B2 shows the mean ratio of the ORs against the quantity of assortative mating for different sample and effect sizes. The results presented here closely resemble those of the linear models. The ratio of ORs for the maternal only and paternal only models tend towards the null (i.e. 1, indicating no difference between the two ORs) as assortative mating increases. The point estimate of the ratio of ORs obtained from the mutually adjusted model is not influenced by assortative mating, however, the confidence interval for the difference increases as a result the maternal and paternal exposure variables reflecting more similar information. This change to the width of the confidence interval is more noticeable at smaller sample sizes than larger sample sizes.


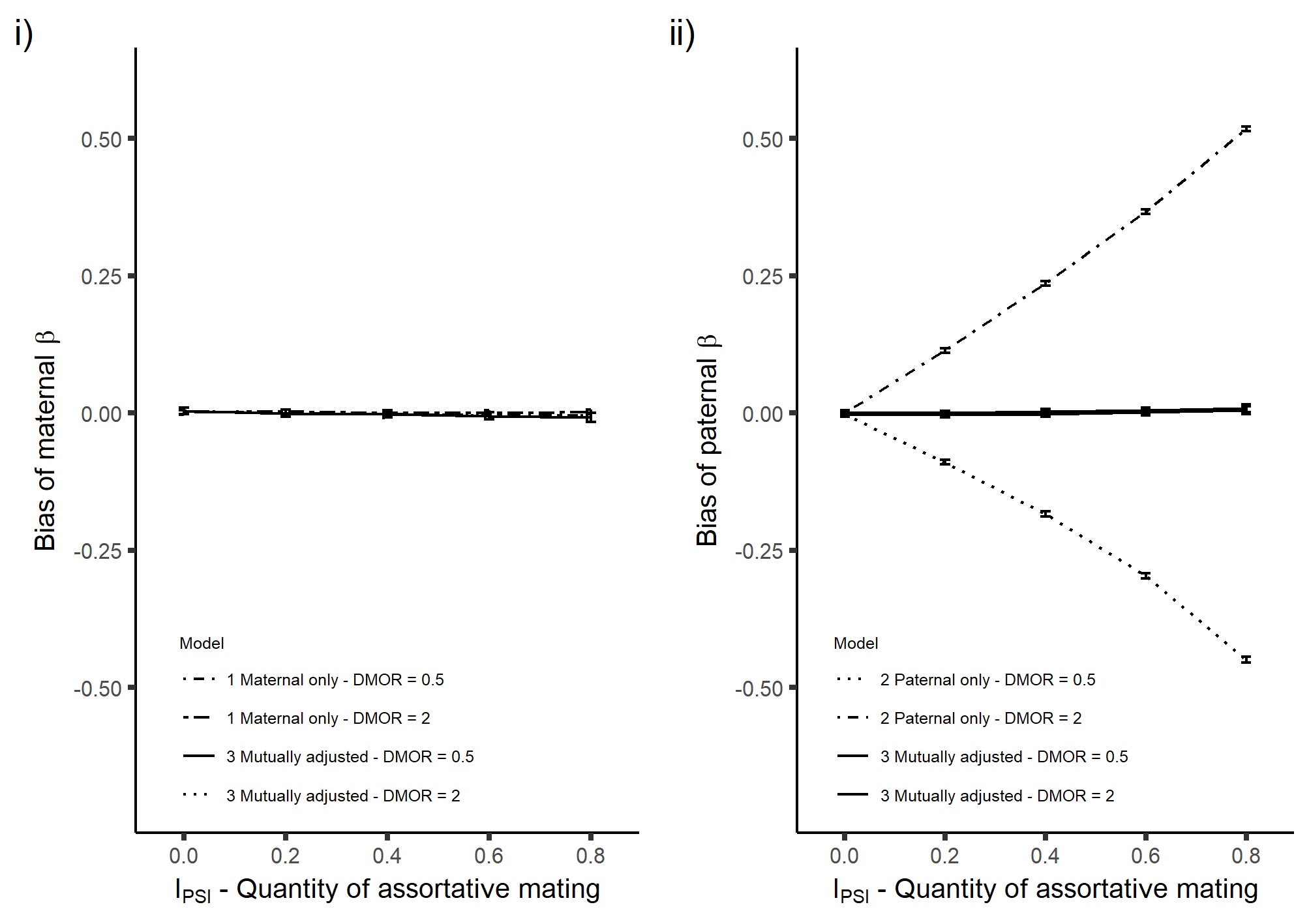


Figure B1: Plots of bias against percentage assortative behaviour for binary outcome data for a) the maternal coefficient and b) the paternal coefficient. Error bars are 95% Monte Carlo confidence intervals across simulations. Sample size for data shown is 10,000. Note the large difference in Y-axis scale between the two plots.


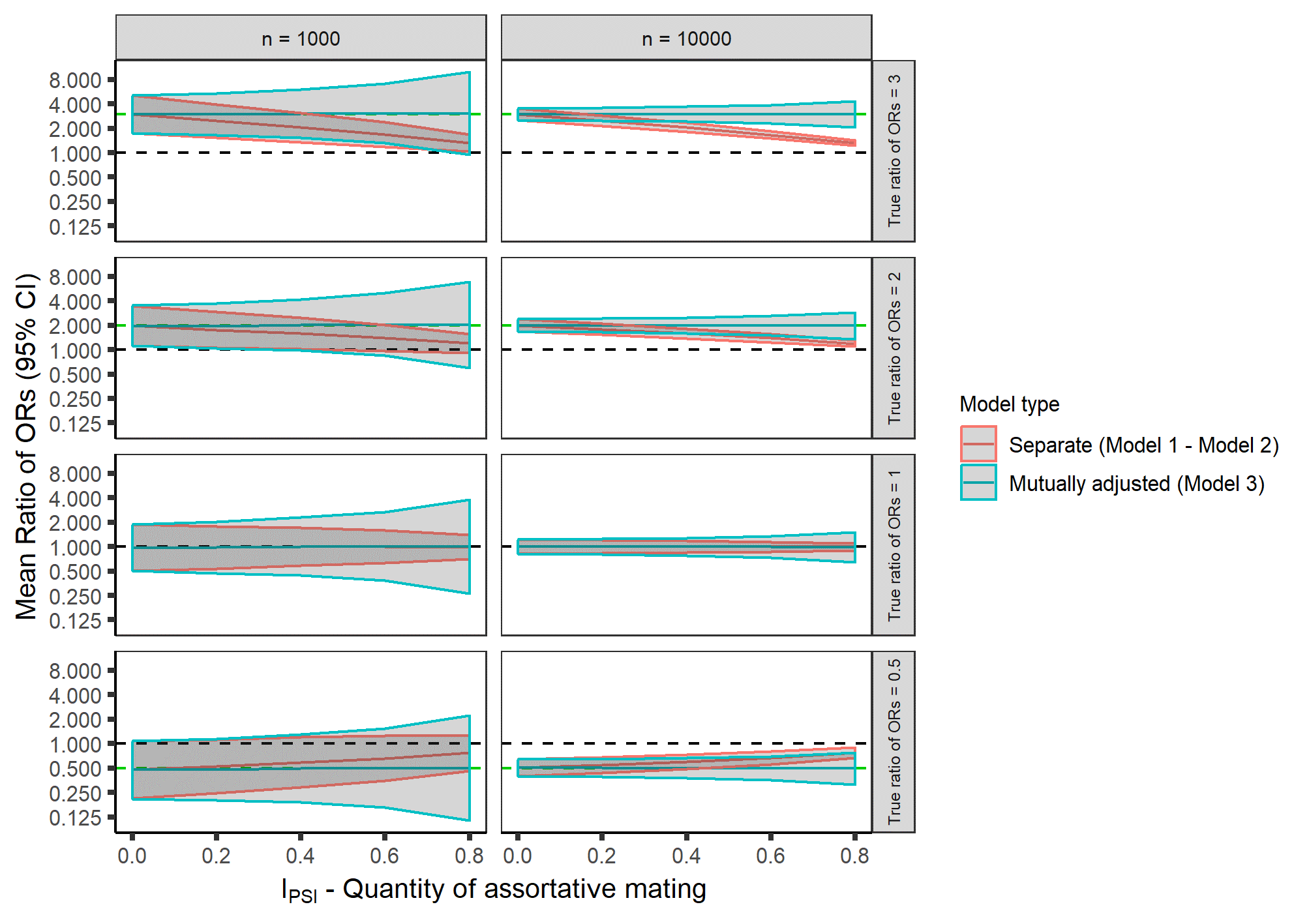


Figure B2: Plot of the mean ratio of ORs across simulations of maternal and paternal β coefficients against the percentage of assortative mating for binary outcome data. 95% confidence bands are the mean lower and upper CI for the difference, produced using bootstrapping. We present the difference between the coefficients of the maternal and paternal only models (red band) and the mutually adjusted model (blue band) for sample sizes of 1000 and 10 000.

# Appendix C: supplementary figures


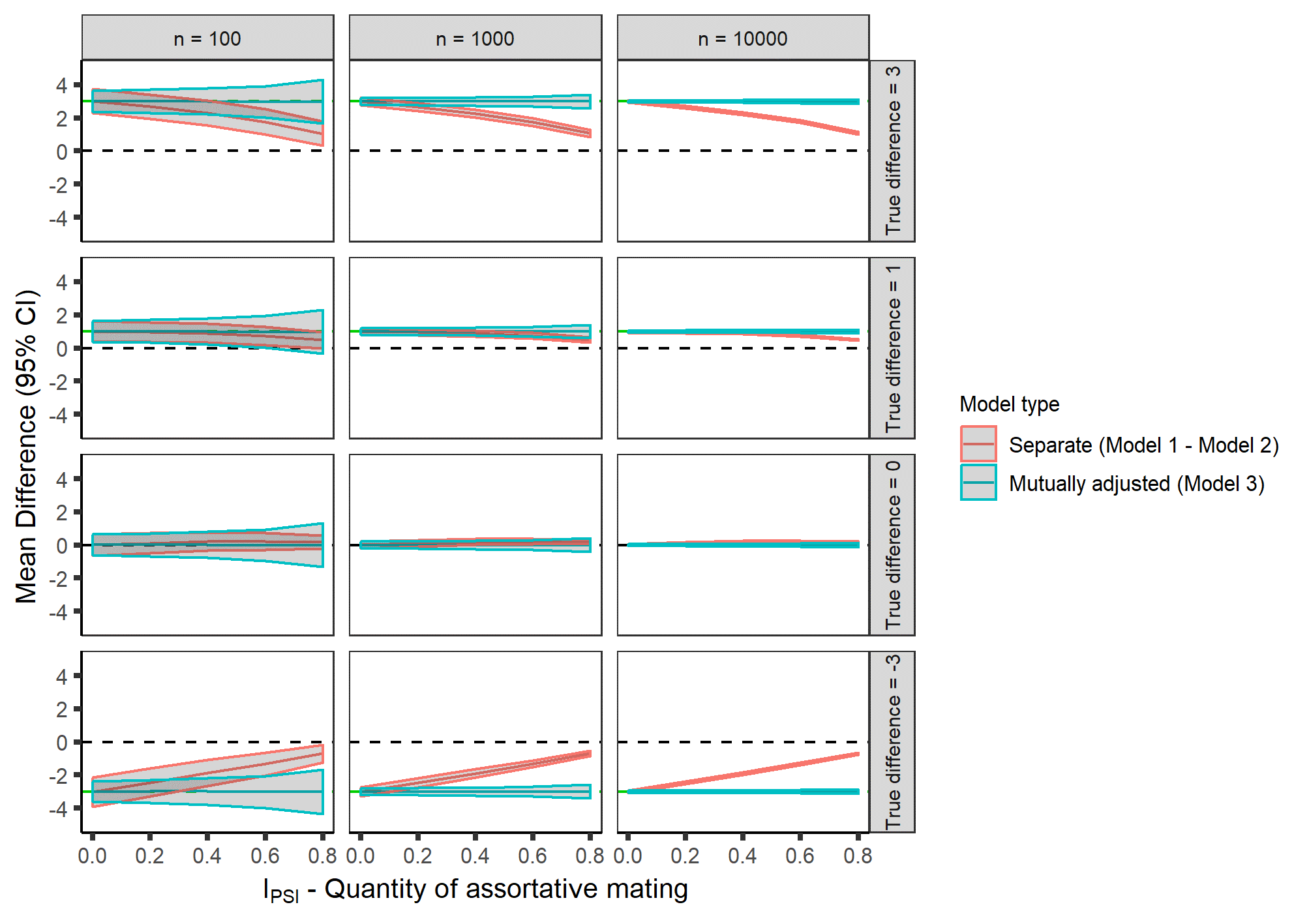


Figure C1: Plot of the mean difference across simulations of maternal and paternal β coefficients against the percentage of assortative mating for data in which maternal and paternal smoking have independent effects. 95% confidence bands are the mean lower and upper CI for the difference, produced using bootstrapping. We present the difference between the coefficients of the maternal and paternal only models (red band) and the mutually adjusted model (blue band) for sample sizes of 100, 1 000 and 10 000.

# Appendix D: applied example of maternal smoking during pregnancy and offspring intellectual disability in the Avon Longitudinal Study of Parents and Children

In our applied example we set out how we would undertake a negative control study to assess the evidence for causality in the association between maternal smoking during pregnancy and offspring intellectual disability. The intended dataset is the Avon Longitudinal Study of Parents and Children (ALSPAC) [3, 4], which recruited 14,541 pregnant women resident in Avon, UK with expected dates of delivery 1st April 1991 to 31st December 1992. Of these pregnancies, there were 13,988 children who were alive at 1 year of age. Please note the study website contains details of all the data that is available through a fully searchable data dictionary (<http://www.bristol.ac.uk/alspac/researchers/our-data/>). For this applied example we replace the term paternal with partner as not all partners in ALSPAC are the biological father of the child. It has been argued that when the exposure is a behaviour, such as smoking, whether the partner is the biological father is not important [5]. Where the exposure is biological, such as BMI, non-paternity may be of greater relevance.

In this study we would use both binary and continuous measures of smoking in pregnancy at 18 weeks gestation based on maternal and partner self-report. There are greater numbers of maternal than partner responders within ALSPAC. Due to high concordance between partner’s self-report and maternal report of partner smoking [6], where possible missing partner values would be imputed using maternal reported values. Number of cigarettes smoked per day was captured using maternal and partner self-report in categories from 0 to 30+ cigarettes in increments of 5. The lowest number from each category would be used to create the continuous measure. For the outcome, a multi-sourced indicator of intellectual disability would be derived using IQ tests at age 8, statements of special educational needs for cognition and learning needs measured at age 11, and NHS linked data. The potential confounding variables were all captured during pregnancy using self-report questionnaires. These variables are parental age at birth, education, social class, financial difficulties, antenatal depression and alcohol use and child parity. It should be noted that all these potentially confounding variables are likely to be more similar within mating pairs than between pairs. A DAG of the assumed relationships between variables is presented in Figure D1.

We would run 4 logistic regression models, repeated unadjusted and adjusted for confounding variables and also using the binary and continuous exposure variables. As before, model 1 would be the maternal only model, model 2 would be the partner only model (included for completeness only) and model 3 would be the mutually adjusted model. We would calculate the difference between the maternal and paternal coefficients in model 3 and use bootstrapping to create a confidence interval. A final model, model 4, would be a mutually adjusted model with an interaction term between maternal and partner coefficient. A likelihood ratio test between model 4 and model 3 can be used to test for an interaction between maternal and partner smoking. The purpose of the additional model is to identify non-linear combinatory effects of exposure and negative exposure and is discussed further later.

Due to the greater participation of mums than their partners the sample size of a complete case analysis may decrease substantially when both maternal and partner cofounding variables are included in analyses. Where confounding variables are selected upon in assortative mating or are strongly correlated with a variable that is selected upon, the maternal and partner values are likely to correlate highly. The value of the maternal confounding variable may then be used as a proxy for the partner variable. Where there is low correlation between maternal and partner confounding variables and both are important predictors of the outcome, both should be included in the set of adjustment variables.

U_M_

U_P_

C_P6_

C_M6_

C_P3_

C_P4_

C_P5_

C_P2_

C_P1_

C_M3_

C_M4_

C_M5_

C_M2_

C_M1_

M

P

S_C_

Y

M – Maternal smoking in pregnancy

P – Partner smoking in pregnancy

Y – Offspring intellectual disability

S_C_ – Mate selection based on confounders

C_M1_ – maternal age

C_M2_ – maternal education

C_M3_ – maternal social class

C_M4_ – maternal depression

C_M5_ – maternal alcohol use

C_M6_ – maternal parity

C_P1_ – partner age

C_P2_ – partner education

C_P3_ – partner social class

C_P4_ – partner depression

C_P5_ – partner alcohol use

C_P6_ – partner number of previous children

U_M_ – unmeasured maternal confounders

U_P_ – unmeasured paternal confounders

Figure D1: DAG of the assumed relationships between variables in our applied example. Variables enclosed in boxes are controlled/adjusted for in models.

# Appendix E: simulation study of a negative control design with assortative mating and error in the negative exposure

## Methods

Measurement error in the exposure or negative exposure has previously been shown to lead to biased effect estimates [7]. We investigated error in the negative exposure in the context of assortative mating. Frequently in a cohort study maternal report of paternal behaviours are used as a proxy for paternal self-report. Where this occurs, there will likely be an increase in the error for the negative exposure but not the exposure.

We repeated part 1 of the simulation study for continuous data only with the addition of error to the negative exposure/paternal smoking value. No error was added to the exposure/maternal smoking value. Again, we did not repeat the simulation study for the binary exposure due to the potential for non-collapsibility to influence our findings. Three types of error were considered: 1) Random error in the paternal smoking value, 2) over-reporting of assortative mating (where mothers report that their partner has the same exposure behaviour more often than is true) and 3) over-reporting of assortative mating among maternal smokers only. The quantities of error considered were 5%, 10% and 20%.

## Results

Figure E1 shows the bias in the paternal coefficient against percentage assortative mating for each error type and error quantity. None of the error types introduces influenced the mutually adjusted models. Randomly adding error to the paternal exposure (error type 1) reduced bias in the paternal only model at larger quantities of assortative mating compared to data without error. The more error the more bias was reduced. This is likely due to the error reducing the correlation between maternal and paternal smoking value.

Models with error type 2 or 3 showed bias that was greater than the models without error. Bias was observed even when there was no assortative mating for models with these types of error. Bias became closer in size to the models without error as the percentage of assortative mating increased.

## Discussion

We showed that error in the negative exposure value, such as when maternal report of a paternal behaviour, can lead to bias in the coefficient estimate for the NCA by making the negative exposure appear to be more or less like the exposure. Mutual adjustment appears to reduce bias occurring in such a way.

By considering error only in the negative exposure we have assumed that maternal report of maternal behaviour is measured with less error than maternal report of paternal behaviour. Prior work on the error rate of maternal report of maternal smoking behaviour has suggested that non-smokers are unlikely to falsely report active smoking [8] while 5% of those reporting that they are non-smokers have been suggested to be current smokers [9, 10]. In contrast, assessment of the agreement between self and maternal reports of paternal smoking in ALSPAC has shown a 5% discrepancy [6]. This does not suggest that the error rate of maternal self-report and maternal report of paternal smoking is equivalent as the 5% error rate for maternal self-report is among non-smokers only. It is also unclear whether those who self-report with error are the same individuals who would report with error for their partners.

Assortment of behaviours may influence the error in the negative exposure. Partners with similar interest in scientific research may be more likely to both engage with cohort studies. Dissimilar interest may lead to one parent engaging with a study while the other does not. Similarity of interest in scientific research may in turn be associated with similarity in behaviours which would be used as negative exposures. As a result, exposure discordant couples may be more likely to rely on maternal report of paternal behaviours. The above patterns of behaviour are speculative and further research is required to identify what patterns of error truly occur for maternal and paternal reports of smoking behaviour and what the determinants of these are. We have, however, provided some insight as to how error could influence conclusions from the negative control design in the presence of assortative mating.


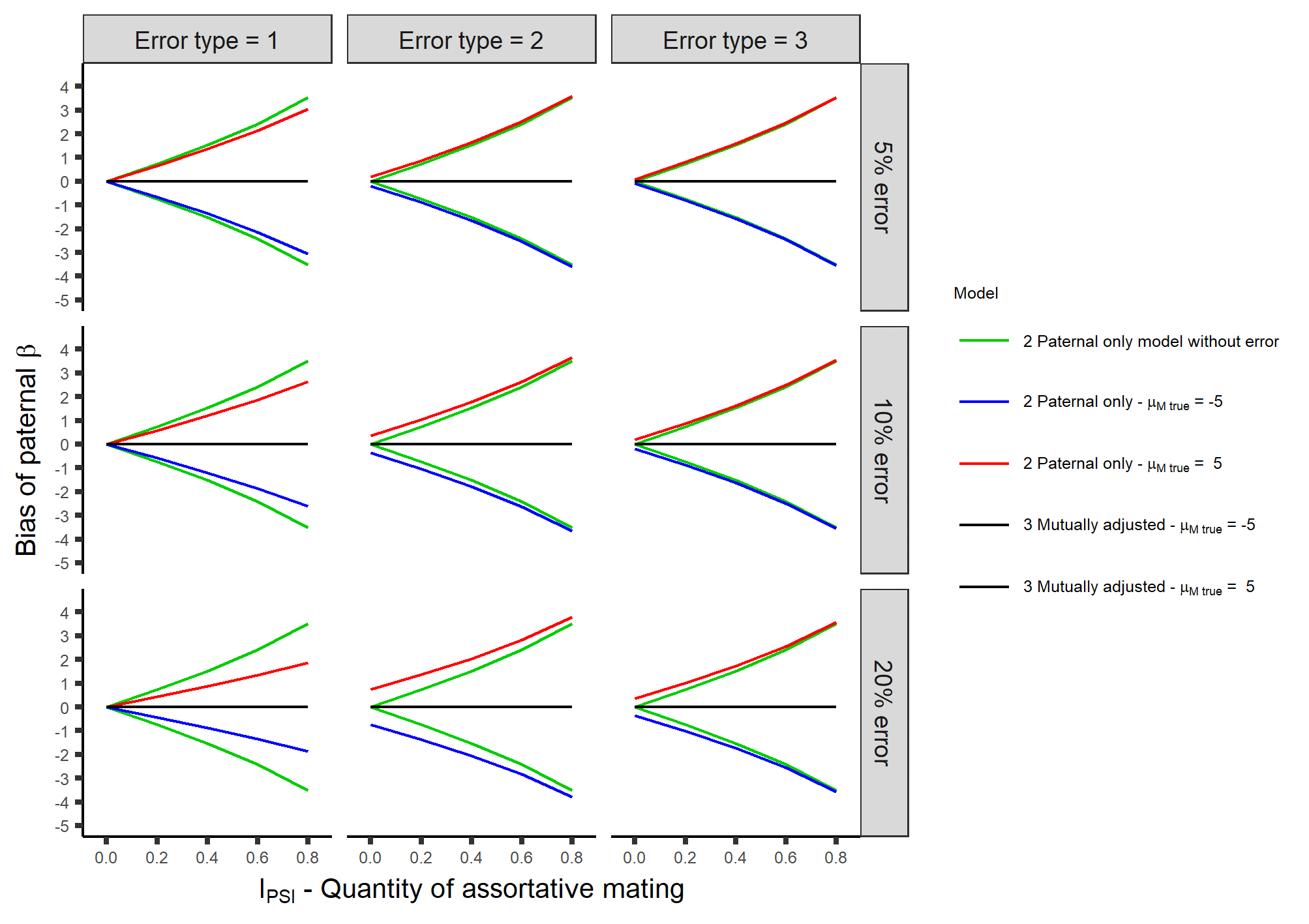


Figure E1: Plot of the bias in the paternal β value against the quantity of assortative mating under three different error structures. Error type 1 is random error to the paternal smoking value, error type 2 is over-reporting of assortative mating (where mothers report that their partner has the same exposure behaviour more often than is true) and error type 3 is over-reporting of assortative mating among maternal smokers only.

# Supplementary material references

1. Rolan-Alvarez, E. and M. Caballero, *Estimating sexual selection and sexual isolation effects from mating frequencies.* Evolution, 2000. **54**(1): p. 30-36.

2. Rolan-Alvarez, E., et al., *The scale-of-choice effect and how estimates of assortative mating in the wild can be biased due to heterogeneous samples.* Evolution, 2015. **69**(7): p. 1845-1857.

3. Boyd, A., et al., *Cohort Profile: the 'children of the 90s'--the index offspring of the Avon Longitudinal Study of Parents and Children.* International journal of epidemiology, 2013. **42**(1): p. 111-27.

4. Fraser, A., et al., *Cohort Profile: the Avon Longitudinal Study of Parents and Children: ALSPAC mothers cohort.* Int J Epidemiol, 2013. **42**(1): p. 97-110.

5. Smith, G.D., *Assessing intrauterine influences on offspring health outcomes: can epidemiological studies yield robust findings?* Basic Clin Pharmacol Toxicol, 2008. **102**(2): p. 245-56.

6. Passaro, K.T., et al., *Agreement between self and partner reports of paternal drinking and smoking. The ALSPAC Study Team. Avon Longitudinal Study of Pregnancy and Childhood.* Int J Epidemiol, 1997. **26**(2): p. 315-20.

7. Sanderson, E., C. Macdonald-Wallis, and G. Davey Smith, *Negative control exposure studies in the presence of measurement error: implications for attempted effect estimate calibration.* Int J Epidemiol, 2018. **47**(2): p. 587-596.

8. Caraballo, R.S., et al., *Factors associated with discrepancies between self-reports on cigarette smoking and measured serum cotinine levels among persons aged 17 years or older: Third National Health and Nutrition Examination Survey, 1988-1994.* Am J Epidemiol, 2001. **153**(8): p. 807-14.

9. Klebanoff, M.A., et al., *Accuracy of self-reported cigarette smoking among pregnant women in the 1990s.* Paediatr Perinat Epidemiol, 2001. **15**(2): p. 140-3.

10. Mattsson, K., et al., *Cotinine Validation of Self-Reported Smoking During Pregnancy in the Swedish Medical Birth Register.* Nicotine Tob Res, 2016. **18**(1): p. 79-83.
